# A Rare and Unusual Evolution of Hypothyroidism in Hashimoto's Thyroiditis to Graves' Disease: A Case Report and Literature Review

**DOI:** 10.7759/cureus.59059

**Published:** 2024-04-26

**Authors:** Amal Essouabni, Fatima Zahrae Melki, Mohammed Amine Essafi, Hayat Aynaou, Houda Salhi

**Affiliations:** 1 Department of Endocrinology, Diabetology, Metabolic Diseases and Nutrition, Hassan II University Hospital, Fez, MAR

**Keywords:** tbab, tsab, trabs, anti-tpo, hyperthyroidism, hypothyroidism, hashimoto's thyroiditis, graves' disease

## Abstract

Our article examines a rare case where hypothyroidism due to Hashimoto's thyroiditis progressed, after a long period (three years) of L-thyroxine substitution, into confirmed hyperthyroidism due to Graves' disease in a 69-year-old man.

The article explores possible mechanisms of this unusual transition based on our case and others reported in the literature. Findings suggest that the coexistence of Hashimoto's thyroiditis and Graves' disease can lead to transitions between hypothyroidism and hyperthyroidism, influenced by the predominance of involved antibodies and residual capacity of thyroid tissue. The authors emphasize the importance of further studies to better understand these transitions and identify at-risk patients. In conclusion, the article highlights the necessity of considering the rare possibility of transition to Graves' disease in patients presenting with persistent hyperthyroidism despite cessation of L-thyroxine.

## Introduction

Hashimoto's thyroiditis and Graves' disease are autoimmune thyroid disorders characterized by distinct pathophysiologies. Recent case reports suggest that they may succeed each other in some individuals [1,2].

The typical scenario involves the evolution from Graves' disease to Hashimoto's thyroiditis as the autoimmune response shifts from thyroid stimulation to blockade [3]. However, the reverse transition is rarely reported in the literature, and its pathogenic mechanism remains poorly understood [4].

In this article, we describe a case of hypothyroidism on Hashimoto's thyroiditis that progressed to Graves' disease after three years of treatment with L-thyroxine, while discussing our case in comparison with available literature data, aiming to describe the mechanism, clinico-biological features, and management modalities of this unusual entity.

## Case presentation

Our patient was a 69-year-old man with the following medical history: slow-onset type 1 diabetes treated with basal-bolus insulin therapy; a secondary adrenal insufficiency, with low adrenocorticotropic hormone (ACTH) and normal hypothalamic-hypophyseal (HH) MRI; asthma, well controlled on Ventolin on demand; in addition, the patient had a family history of Graves' disease in a sister.

He was followed for three years for hypothyroidism due to Hashimoto's thyroiditis, discovered as part of screening for autoimmune diseases in type 1 diabetics.

Initially, the patient presented with asthenia and recurrent hypoglycemia despite insulin adjustment, without other signs of hypothyroidism.

The cervical examination did not detect a goiter. The etiological assessment revealed positive anti-thyroid peroxidase (anti-TPO) at 715 U/ml, with cervical ultrasound showing a small-volume thyroid, hypervascularized without high flow, and coarse echostructure consistent with thyroiditis. The patient was started on L-thyroxine substitution therapy at 50 ug/day with good clinical and biological improvement.

After three years of treatment, the patient developed hyperthyroidism: ultrasensitive thyroid-stimulating hormone (TSHus) = 0.01 uUI/ml; T4 = 10.59 pg/ml (6-11.2); T3 = 3.35 (2.5-3.9). Despite discontinuation of treatment, hyperthyroidism persisted and worsened (Table 1).

**Table 1:** Evolution kinetics of laboratory tests in our patient. TSHus: ultrasensitive thyroid-stimulating hormone; T4: thyroxine; T3: triiodothyronine; TRAK: thyroid-stimulating hormone receptor antibodies.

	04/19	10/19	01/20	05/20	07/20	12/20	01/21	12/21	02/22
TSHus (uUI/ml)	1.98	0.1	0.02	0.01	Not done	0.009	Not done	9.16	0.004
T4 (pg/ml)	Not done	Not done	Not done	10.59 (6-11.2)	10.29 (6-11.2)	21.64 (13-21)	6.10 (7-14)	8.8 (7-14.8)	15.6 (1.05 times the normal level)
T3 (pg/ml)	Not done	Not done	Not done	3.35 (2.5-3.9)	Not done	Not done	2.04 (1.7-3.7)	2.03 (1.7-3.7)	4.58 (1.23 times the normal level)
Antithyroperoxidase antibodies (UI/mL)	715	Not done	Not done	Not done	78.66	Not done	Not done	Not done	Not done
TRAK (UI/ml)	Not done	Not done	Not done	Not done	6.2 (6 times the normal level)	Not done	Not done	Not done	14.7 ui/l (5 times the normal level)
Treatment	L-thyroxine 50ug/j	Decrease to 25 ug/day	Discontinuation of L-thyroxine	Stopping treatment	Stopping treatment	Dimazol 10 mg/j	Dimazol 10 mg/j	Dimazol 10 mg	Stopping treatment

Clinical examination revealed tachycardia at 101 bpm, with a normal cervical examination, notably no goiter, nodule, or palpable adenopathy, and no exophthalmos.

Given this presentation, an etiological assessment suggested Graves' disease: thyroid-stimulating hormone receptor antibodies (TRAKs) at six times the normal level and persistent antithyroperoxidase antibody positivity (78.66 IU/mL).

Cervical ultrasound revealed a thyroid gland of normal size (right lobe: 4.38 ml, left lobe: 5.6 ml, isthmus: 3.7 mm), hypervascularized, with a heterogeneous hypoechoic echostructure interspersed with hyperechoic septations, compatible with thyroiditis.

**Figure 1:**
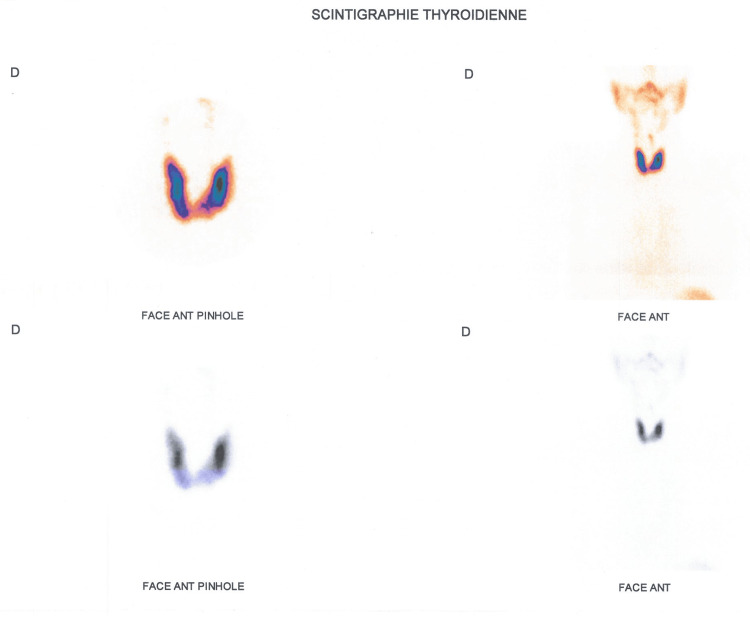
Thyroid scintigraphy image showing a thyroid gland of normal size, with regular contours, intense and homogeneous uptake, without clearly identifiable nodular formation, suggestive of Graves' disease.

Thyroid scintigraphy (Figure 1) confirmed Graves' disease, and the patient was treated with a synthetic antithyroid: carbimazole 10 mg for two years.

Given the failure of medical treatment (persistent hyperthyroidism and TRAKs positive at five times the normal level), radioiodine therapy was indicated. Six weeks after the treatment, the patient developed hypothyroidism, which was successfully treated with substitutive L-thyroxine.

## Discussion

In this article, we have examined a rare case of unusual progression from hypothyroidism on Hashimoto's thyroiditis to hyperthyroidism on Graves' disease after three years on L-thyroxine in a 69-year-old individual. This case required radical treatment with radioiodine therapy after failure of medical treatment with synthetic antithyroid drugs (STDs).

Graves' disease and Hashimoto's thyroiditis both share an autoimmune pathogenesis. Although rare, these pathologies can coexist [5]. Between 50% and 70% of patients with Graves' disease simultaneously present with anti-thyroid peroxidase (anti-TPO) antibodies and/or anti-thyroglobulin (anti-Tg) antibodies, indicating concomitant Hashimoto's thyroiditis [6,7].

In this case, the positivity of these antibodies (anti-TPO and anti-Tg) indicates an increased risk of progression to hypothyroidism due to Hashimoto's thyroiditis [8]. This scenario represents the progression most frequently observed in the literature (approximately 15% to 20%) [9].

The reverse conversion (from hypothyroidism due to Hashimoto's thyroiditis to Graves' disease) remains a rare clinical situation and is rarely described in the literature [3,10,11]. To our knowledge, only 50 cases have been reported [9].

The rarity of this conversion is thought to be due to the critical and irreversible loss of functional thyroid mass that can react to thyrotropin receptor antibody (TRAb), through prolonged destructive action of anti-TPO and anti-Tg antibodies [12].

The first was described for the first time in 1968 by Wyse and colleagues [13]. However, to date, the exact pathogenic mechanism remains poorly understood [3].

Among the explanations described in the literature for this transition is the simultaneous presence in the same individual of two types of antibodies acting on the thyroid-stimulating hormone (TSH) receptor with different effects on thyroid cells [14]. On the one hand, TSH receptor-stimulating antibodies (TSAb) are responsible for Graves' disease. On the other hand, TSH receptor-inhibiting antibodies (TBAb) rarely cause hypothyroidism [15].

In this case, thyroid function may oscillate between hypothyroidism and hyperthyroidism, depending on the predominance of stimulating (TSAb) or blocking (TBAb) receptors [16].

This is demonstrated by the study by Takasu et al. of 34 known TBAb-positive patients with hypothyroidism, which revealed a disappearance of TBAb in 15 of the 34 patients over a 10-year period. While two patients developed TSAb-positive Graves' hyperthyroidism. This suggests that TBAb-positive hypothyroidism and TSAb-positive hyperthyroidism may be two facets of the same disease, i.e., TRAb disease [16].

However, this explanation remains insufficient to fully understand the transition from hypothyroidism due to Hashimoto's thyroiditis to Graves' disease. This is because the mechanism of hypothyroidism induced by TBAb differs from that of Hashimoto's thyroiditis. In the latter, hypothyroidism results from the destruction of thyrocytes caused by antibodies directed against specific antigens such as thyroid peroxidase and thyroglobulin, leading to inflammation, cell destruction, necrosis, and apoptosis of follicular cells, followed by fibrosis [8].

The retrospective study by Gonzalez-Aguilera et al. of 24 patients with Hashimoto's thyroiditis who subsequently developed Graves' disease after approximately 38 ± 45 months found that thyroid volume in these patients ranged from 3.1 to 12.3 ml. There was no significant difference in thyroid volume at the time of diagnosis of Hashimoto's thyroiditis compared to that of Graves' disease [3].

A potential explanation for this transition is that the autoimmune tissue lesions, which initially cause significant thyroid underactivity, may partially recover over time, thereby allowing subsequent stimulation by TSAb [16,17].

Our patient also exhibited a normal thyroid volume during hyperthyroidism despite a long duration of evolution from hypothyroidism due to Hashimoto's thyroiditis, thus confirming effective stimulation by TRAbs, as observed in the previously mentioned study.

However, it is possible that hypothyroidism in our patient may not be solely attributable to major destruction by anti-TPO, but rather to an association with simultaneous inhibitory activity of TBAb.

This coexistence could explain the absence of major gland destruction despite proven and prolonged hypothyroidism.

Furthermore, it is conceivable that our patient simultaneously had antibodies from both Graves' disease (TSAb and TBAb) and Hashimoto's thyroiditis, with an initial predominance of TBAb, anti-TPO, and anti-Tg responsible for hypothyroidism. However, we are limited in confirming this hypothesis as TBAb and TSAb were not initially measured due to the absence of an apparent cause justifying their measurement in this patient.

The association between these different antibodies has been described in the literature, for example, in a study of 87 patients with Hashimoto's thyroiditis, positive values of TSAb were found in 10% of cases, and TBAb in 11% [18].

Also, Horiya et al. described in 2020 a case of a 44-year-old woman who had Graves' disease with all four positive antibodies (TSAb, TBAb, anti-TPO, and anti-Tg). Furthermore, the histopathological examination revealed both characteristics of Graves' disease and Hashimoto's thyroiditis at the same time. Based on these results, she was diagnosed with both diseases at the same time (Graves' disease and Hashimoto's thyroiditis) [19].

It is possible that our patient also had a coexistence of these two pathologies, with an initial predominance of TBAb, anti-TPO, and anti-Tg antibodies.

In this context, a case was published by Narantsatsral Daramjav et al. in 2023 of a 50-year-old woman initially treated for hypothyroidism, who presented with high levels of anti-TPO, anti-Tg, and TSAb, with negative TRAb. After five weeks of levothyroxine treatment, she developed persistent hyperthyroidism, even after two months of discontinuing the medication. Serum thyroglobulin levels were normal, ruling out iatrogenic hyperthyroidism or destructive thyroiditis. TSAb, anti-TPO, and anti-Tg antibodies remained positive, confirming the diagnosis of Graves' disease, with a favorable response to synthetic antithyroid drugs [9].

Based on our case and cases reported in the literature, it is concluded that Hashimoto's thyroiditis and Graves' disease can coexist with a possible transition from hypothyroidism to hyperthyroidism and vice versa, depending on the predominant antibody type, and also on the residual functional capacity of the thyroid tissue after a phase of hypothyroidism due to the destructive action of anti-TPO and anti-Tg.

In these cases, alternating episodes of hypothyroidism and hyperthyroidism can be stopped by thyroidectomy or radioactive thyroid ablation [20,21].

In the end, to fully understand the exact mechanism of transition from Hashimoto's thyroiditis to Graves' disease, we still need studies on larger samples, including measurement of the various autoantibodies, as well as immunological and genetic studies, with precise analysis of the various parameters that can influence this conversion. This will help to better understand the pathophysiology and also assist in identifying patients at increased risk of transitioning to Graves' disease.

## Conclusions

Our clinical case highlights the importance of considering the possibility of a rare transition from hypothyroidism associated with Hashimoto's thyroiditis to Graves' disease in patients on levothyroxine. Although excessive overdosing with levothyroxine is often initially considered in the presence of clinical and biological hyperthyroidism, the persistence of hyperthyroidism after discontinuation of L-thyroxine should raise further suspicion.

Although several hypothetical mechanisms have been proposed to explain this exceptional transition, further immunological and genetic cohort studies are needed to confirm these hypotheses.
